# A Complicated Case of Vertebral Osteomyelitis by Serratia Marcescens

**DOI:** 10.7759/cureus.9002

**Published:** 2020-07-04

**Authors:** Abdul Rana, Noor Ul Ann Rabbani, Samuel Wood, Colin McCorkle, Christine Gilkerson

**Affiliations:** 1 Internal Medicine, Marshall University Joan C. Edwards School of Medicine, Huntington, USA

**Keywords:** vertebral osteomyelitis, serratia marcescens, hematogenous, intravenous drug abuse

## Abstract

Osteomyelitis is an infection of the bone and bone marrow that can be caused by an exogenous source or by hematogenous spread. The hematogenous spread of bacteria usually results in bacteremia with subsequent infection of the bone. The most commonly associated pathogen with this type of infection is Staphylococcus aureus, with other gram-negative organisms, such as Pseudomonas aeruginosa, also frequently encountered. The microorganism Serratia marcescens is a rare and infrequently encountered cause of this condition known to cause nosocomial infections. This organism can be notoriously difficult to treat, with resistance to many commonly used antibiotics. The case presented is one of vertebral osteomyelitis in an intravenous drug user caused by Serratia marcescens with subsequent treatment and management of the condition. This case allows for investigation into the continued management of intravenous drug user infections, with the isolation and treatment of less commonly encountered pathogens.

## Introduction

Osteomyelitis is an infection localized to the bone. The most common pathogens associated with this infection are Staphylococcus aureus and aerobic gram-negative bacilli. The causes of osteomyelitis can be divided into pyogenic and non-pyogenic, and the most common organisms in each group are Staphylococcus aureus and Mycobacterium tuberculosis, respectively [1]. Osteomyelitis can also be divided based on the mode of spread into the hematogenous and non-hematogenous routes. Serratia species, although well-known, are not common pathogens associated with either classification of osteomyelitis. Serratia is a gram-negative, motile, facultative anaerobic bacillus of the Enterobacteriaceae group. These bacteria are known to cause a spectrum of clinical diseases in humans most often related to indwelling catheters or respiratory infections but can include other infections such as osteomyelitis, meningitis, or septic arthritis. Serratia marcescens has been a well-known cause of severe infections in humans since the 1960s [2]. Currently, it accounts for approximately 2% of nosocomial infections [3]. We present a case of vertebral osteomyelitis caused by Serratia marcescens in an immunocompetent intravenous (IV) drug user and the subsequent treatment and management of his condition.

## Case presentation

**Figure 1:**
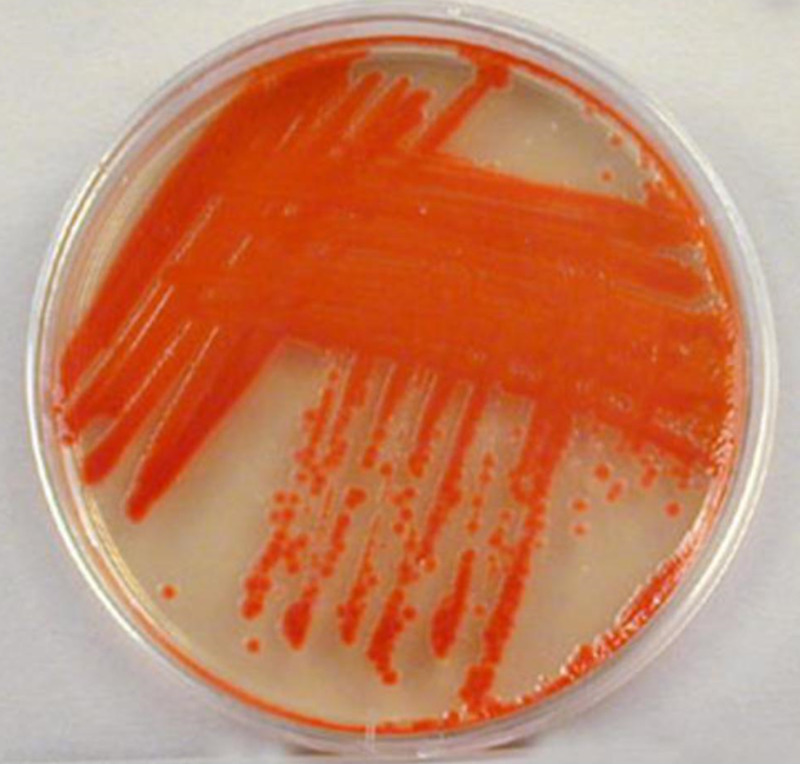
Cultures isolated from the epidural abscess grew Serratia marcescens on blood agar Medium-sized colonies appearing buff in color. Bacteria produce red pigment.

A 29-year-old male with a history of IV drug use, without any known comorbidities presented to the hospital with complaints of neck and back pain for three weeks. He denied any fever or chills, any radiation of pain, or any numbness or tingling. He was seen earlier in the emergency room during that week, given a shot of IV ketorolac for pain, and then discharged. The patient's physical exam was unremarkable for any focal neurological deficits. His illness did not subside, the pain worsened, and he subsequently presented later in the week with worsening neck and back pain. He also complained of an abscess on his right leg, located above the lateral malleolus with clean edges that he first noticed two to three weeks ago. At the time of presentation, he was alert and oriented, and vitals were within normal limits. The computed tomography (CT) scan revealed osteomyelitis at the cervical vertebra (C4-C5) level and prevertebral fluid collection. Neurosurgery was consulted, and magnetic resonance imaging (MRI) was recommended, which revealed C4-C5 osteomyelitis with an epidural abscess. The patient was started on IV vancomycin and admitted to the floor. During his hospital stay, neurosurgery performed an anterior corpectomy and posterior cervical instrumented fusion. The procedure was complicated by a cerebrospinal fluid (CSF) leak, and intraoperative cultures grew Serratia marcescens, as shown in Figure 1. The patient’s hospital course was complicated by the CSF leak and drainage from the surgical wound, for which he had to be taken back to the operating room (OR) for an anterior washout. A lumbar drain was placed that day for drainage. Infectious disease recommended adding aztreonam 2 g IV every eight hours for six weeks. Vancomycin was discontinued after the cultures did not grow methicillin-resistant Staphylococcus aureus (MRSA). Blood cultures were also negative during his hospital stay. The patient completed six weeks of treatment with IV aztreonam and was discharged home after completion of the antibiotics course. He was followed up in the clinic where his condition has significantly improved and he did not have any active complaints during that visit.

## Discussion

Serratia is an infrequent cause of osteomyelitis that is usually seen in immunocompromised patients [3]. Serratia marcescens is a motile, gram-negative bacillus from the Enterobacteriaceae family, with natural red pigment. It has been associated with nosocomial opportunistic infection and intravenous drug abuse (IVDA) [2,4]. Although frequently known for its pigment production, one study found the non-pigment producing strands to be most often isolated in the hospital setting, mainly infecting immunocompromised hosts [5]. Serratia marcescens was discovered by an Italian pharmacist in 1819 and produces beta-lactamase that can make it challenging to treat. Some of the risk factors associated with this infection are due to instrumentation or catheterization. Serratia has various virulent features that include hemolysin production, swarming, and biofilm production, which are regulated by RssAB signaling and temperature [4]. Although this particular species of Serratia is a rare cause of osteomyelitis, it is not unheard of even in a patient with no predisposing factors [5]. Vertebral osteomyelitis is a rare form of osteomyelitis, accounting for 2%-7% of all cases, and has an incidence rate of 2.4 cases per 100,000 [1]. Historically, the classic pathogen associated with pyogenic vertebral osteomyelitis is Staphylococcus aureus, both the methicillin-resistant and methicillin-sensitive strains.

Our patient likely developed hematogenous osteomyelitis by direct inoculation associated with IV drug use, which then subsequently spread to the C4-C5 area of the spine [2]. He had a history of IVDA, which predisposed him to this particular infection. Bacterial cultures are essential to form a diagnosis. Serratia resides in water, making hospital sinks a potential source, and has also been known to be spread by healthcare workers [5]. There is widespread antibiotic resistance to Serratia marcescens due to the production of beta-lactamase [3,6]. The drug of choice should be tailored according to the site of infection and the specificity and sensitivity of the organism to a particular antibiotic. Serratia has been susceptible to numerous antibiotics such as third and fourth generation cephalosporins, aztreonam, carbapenem, and trimethoprim-sulfamethoxazole. Serratia has been found to be resistant to colistin and cephalothin [7].

The fact that this patient had a history of Intravenous drug abuse predisposed him to vertebral osteomyelitis, but the causative organism being Serratia is not entirely unknown but somewhat unusual. There is limited data regarding the prevalence of community-acquired Serratia infections. Still, there have been well-documented reservoirs of Serratia in the hospital setting, along with outbreaks of Serratia related to a contaminated source [8]. A study from Blossom et al. found Serratia sources in saline solutions and pre-filled heparin syringes [9]. A survey from Bopp DJ et al. found a Serratia outbreak related to contaminated IV magnesium sulfate [10]. These studies could suggest a similar possible growth to our presented case, possibly that the current patient had a source of Serratia on the IV syringe for drug abuse.

## Conclusions

The case presented offers a unique opportunity to explore the relationship between IV drug use and osteomyelitis with a rare pathogen. This study could be extended further by analyzing other, related infectious sources of Serratia marcescens to look for shared epidemiological features.
